# The association between opening a short stay paediatric assessment unit and trends in short stay hospital admissions

**DOI:** 10.1186/s12913-021-06541-x

**Published:** 2021-05-29

**Authors:** Steve Turner, Edwin-Amalraj Raja

**Affiliations:** 1 Child Health, University of Aberdeen, Aberdeen, UK; 2 Women and Children Division, NHS Grampian, Aberdeen, AB25 2ZG UK; 3 Medical Statistics Team, University of Aberdeen, Aberdeen, UK

**Keywords:** Child, Epidemiology, Health services, Hospitalization

## Abstract

**Background:**

Many inpatient facilities in Scotland have opened short stay paediatric assessment units (SSPAU) which are clinical areas separate from the usual inpatient ward area and these are where most short stay (also called zero day) admissions are accommodated. Here we describe the effect of opening short stay paediatric assessment units (SSPAU) on the proportion of zero day admissions relative to all emergency admissions.

**Methods:**

Details of all emergency medical paediatric admissions to Scottish hospitals between 2000 and 2013 were obtained, including the number of zero day admissions per month and health board (i.e. geographic region). The month and year that an SSPAU opened in each health board was provided by local clinicians.

**Results:**

SSPAUs opened in 7 health boards, between 2004 and 2012. Health boards with an SSPAU had a slower rise in zero day admissions compared to those without SSPAU (0.6% per month [95% CI 0.04, 0.09]. Across all 7 health boards, opening an SSPAU was associated with a 13% [95% CI 10, 15] increase in the proportion of zero day admissions. When considered individually, zero day admissions rose in four health boards after their SSPAU opened, were unchanged in one and fell in two health boards. Independent of SSPAUs opening, there was an increase in the proportion of all admissions which were zero day admissions (0.1% per month), and this accelerated after SSPAUs opened.

**Conclusion:**

Opening an SSPAU has heterogeneous outcomes on the proportion of zero day admissions in different settings. Zero day admissions could be reduced in some health boards by understanding differences in clinical referral pathways between health boards with contrasting trends in zero day admissions after their SSPAU opens.

**Supplementary Information:**

The online version contains supplementary material available at 10.1186/s12913-021-06541-x.

## Background

In the United Kingdom there are no primary care paediatricians, and care in the community is provided by family doctors who refer acutely unwell children to the hospital if care in the community is not felt to be in the child’s best interests. Short stay paediatric assessment units (SSPAU) are clinical areas in hospital where children can be observed for a period of up to 24 h during which a decision can be made to admit them to a medical ward for ongoing in patient management or discharged home [1]. SSPAUs are found in many countries including the Unites States of America [2], Australia [3] and the UK [1]. Many hospitals in the UK opened SSPAUs, beginning in the 1990s and there are many benefits to SSPAU including a faster turnover of patients and an improved patient and family experience [1].

The impact of opening an SSPAU on the number of admissions is not clear. A systematic review of the literature published in 2005 [4] suggested that SSPAUs might be effective in reducing hospital admissions, but a later review [5] concluded that the evidence underpinning the earlier review [4] was not sufficiently robust to support any firm conclusion. A systematic review of studies from the USA found no significant change in admission numbers after the opening of observation units (similar to SSPAUs), but did cite individual studies which found reduced admissions for some specific conditions including asthma and gastroenteritis after an observation unit opened [2]. An Australian study found that opening a Children’s Emergency Annexe reduced the number of bed days and reduced costs [3]. A reduction in admissions associated with opening SSPAUs may be simply due to recategorising clinical presentations, for example a “reduction” might not include a number of children assessed and sent home.

In the UK there has been a rise in unscheduled medical paediatric admissions, [6–8] which is mostly due to a sharp rise in admissions where the child is admitted and discharged on the same day (called zero day admissions) [7, 9]. In Scotland there was a rise of 13,681 in all unscheduled admissions between 2000 and 2013, and this rise included 13,470 zero day admissions; only 211 admissions lasted more than 24 h [7].

The large majority of zero day admissions occur on SSPAUs, with some occurring in other clinical areas. What is not clear is whether opening SSPAUs in the UK has been the cause or effect of the rise in zero day admissions, and insight into this issue could inform service development and policy.

## Methods

### Aims

The aim of this study was to use the natural experiment of SSPAUs opening in hospitals across Scotland to determine whether zero day admissions (as a proportion of all acute admissions) rose after an SSPAU opened. We also compared trends in zero day admissions over time between regions which had an SSPAU and those that had no SSPAU. Since the number of non-zero day admissions was essentially static between 2000 and 2013, a rise in proportion of zero day admission is approximate to an absolute increase in zero day admissions [7].

### Healthcare setting

**Fig. 1:**
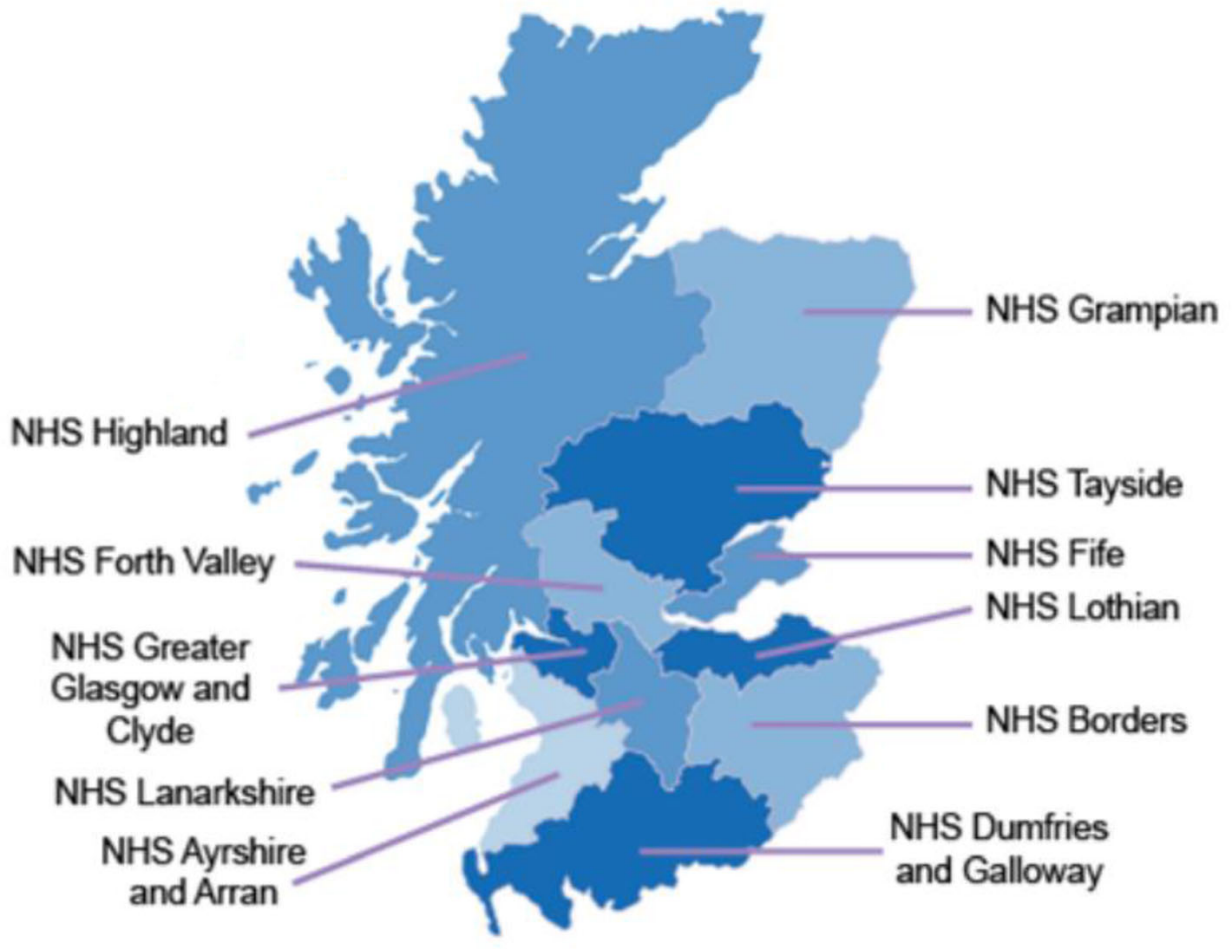
A map showing the location of the eleven NHS health boards in Scotland with acute paediatric inpatient facilities

In the UK (including Scotland), children are admitted to hospital by one of three routes: first, family doctors (general practitioners, GPs) who are based in the community and work 8 am-6 pm Monday to Friday; second, out-of-hours staff who provide care in the community outside of GP working hours and who may be doctors but also include individuals may be relatively inexperienced when it comes to medical decision-making for sick children, e.g. physician assistants; third, emergency department medical staff who assess patients who present directly to hospital. The decision to refer is entirely at the discretion of the GP, member of the out-of-hours team and emergency department clinician. There are 14 regional health boards in Scotland, each of which covers a distinct geographical region, of whom 11 at the time of writing had at least one hospital with acute paediatric medical admitting facilities (Fig. 1).

### Operational definitions

A health board is a geographical area where a single organisation provides healthcare for the population. An SSPAU is usually a clinical area (ward) which is separate from the emergency department and main paediatric inpatient ward, although in some cases the SSPAU can be an area within the main paediatric inpatient ward. The SSPAU is usually staffed by different medical and nursing teams to those on the main paediatric ward, although in evenings and weekend medical staff usually work across SSPAU and the main paediatric ward. Children may be referred to the SSPAU by colleagues working in primary care (including "in hours" and " out of hours" teams comprised of general practitioners, advanced nurse practitioners and physician assistants) and the emergency department. After assessment on the SSPAU children may be discharged home, observed on the SSPAU for a period of up to 24 h or admitted to another inpatient ward (e.g. the medical paediatric ward, high dependency unit) if the admission is likely to last more than 24 h. An admission is defined as an inpatient episode where unscheduled care was provided outside an emergency department setting by medical paediatric staff.

### Study design

This was an observational study describing the proportion of zero day unscheduled (also known as emergency) admissions per month (relative to all acute  unscheduled admissions) before and after an SSPAU was opened in hospitals across Scotland. The primary outcome was the proportion of zero day admissions of all unscheduled admissions per month. Local clinicians were asked if the hospital(s) in their health board had a facility which fitted the description of an SSPAU [1], and if so what month and year the SSPAU opened. Clinicians confirmed that an admission to SSPAU was categorised as an admission in their health board (alternatively as SSPAU admission could be considered akin to an emergency department attendance and not coded as a hospital admission) and that the definition of an admission did not change over the period of interest. Details of the SSPAUs, including number of beds and cubicles and location, were also provided (see the supplement). There were two hospitals in some health board regions and it was not possible to separate admissions to each hospital. Our approach was to analyse data for the whole region as if there was just one hospital and the data  from the SSPAU in the larger hospital was used for the analysis (regardless of whether or not the smaller unit also had an SSPAU) If the larger hospital did not have an SSPAU but the smaller unit did, then the health board was considered not to have an SSPAU. Ethical approval was obtained from the North of Scotland Research Ethics Committee (14/NS/1071).

### Admission data

The details of every admission to hospital in Scotland of individuals aged < 16 years between 2000 and 2013 were provided by the Information Services Division of the Scottish Government [7]. Information from planned admissions was excluded from the present analysis. A zero day admission was defined as an admission with zero days length of stay (i.e. the admission and discharge took place on the same date) and where there was no readmission within the same calendar month. The latter criterion excluded cases discharged home but given instructions to return to hospital if the caregiver was worried (“safety netting”, a common practice on discharging patients). The full list of details are described previously [10] and are also presented in the supplement.

### Analysis

The proportion of zero day admissions and rate of change in proportion of zero-day admission was compared between centres who opened SSPAU and centres who didn’t open during the study period. SSPAUs opened at different points of time, and zero admission and overall admissions per month were referenced to the opening of SSPAU; therefore the opening of SSPAU did not have a specific calendar time in the analysis. An impact model considered whether there was a change in the trend in zero day admissions after an SSPAU was opened. The mean (standard deviation) zero day admissions/month before and after opening SSPAU and the percentage of zero day admissions relative to all admissions/month were calculated. A segmented quasi-Poisson regression analysis of interrupted time-series [11] was performed to analyse time-series trends in zero day admission before and after SSPAU opening. The overall trend in zero day admissions between 2000 and 2013 and the “step change” (i.e. the change seen instantly at the time the SSPAU was opened) are presented. An interaction term between time and opening of SSPAU was used to find the change in the trend in zero day admissions after opening SSPAU. To account for over-dispersion (i.e. variance greater than mean), a scaling adjustment was made to allow the variance to be proportional rather than equal to the mean. Seasonality of admissions was adjusted for through a Fourier term (pairs of sine and cosine functions) [12]. A plot of residual against time was used to examine autocorrelation. Incidence rate ratio was expressed in terms of relative percentage with 95% confidence intervals. Visual inspection of trends in some individual centres revealed non-linear trends in the initial months of data collection, i.e. many months before the SSPAU opened, and these initial months were excluded in sensitivity analyses. As an additional sensitivity analysis, for each individual centre and for all centres combined, data from 24 months before and after the SSPAU opened were analysed to give a focus on changes in admissions with a focus on the period around SSPAU opening. A *p*-value less than 0.05 was considered to be statistically significant. Stata MP ver 15 was used for statistical analysis.

## Results

### Details of SSPAU and zero day admissions

Table 1 presents details of the eleven health boards including population, population density, deprivation index and details of any SSPAU opening. Across all eleven health boards, there were 507,403 acute medical admissions between 2000 and 2013, of which 220,517 (43%) were zero day admissions and an annual incidence of 22/1000 children [7].

**Table 1:** Characteristics of the populations in each health board (a geographical area where a single organisation provides healthcare for the population). The three Scottish NHS boards with no paediatric in patient facility are not included in this table (NHS Orkney, NHS Shetland and NHS Western Isles)

NHS health board	Population of children in 2013 (aged < 15 years^a^)	Area covered by NHS Health board in square miles (children/square mile)	Median deprivation quintile (IQR, 1 = most deprived))	Median age at admission (SD)	Health board details (number of hospitals with medical paediatric ward, when/if SSPAU opened)	Included in analysis	Details of short stay paediatric assessment units (SSPAU)
NHS Ayrshire and Arran	58,660	1310 (45)	2 (1,4)	2.4 (0.8, 7.2)	One medical paediatric ward. SSPAU opened July 2006.	Yes	One hospital, in Kilmarnock where the SSPAU is next to but separate from the inpatient ward. The SSPAU has 10 beds/cots in two cubicles.
NHS Borders	17,756	1831 (15)	3 (2,4)	2.9 (1.0, 8.4)	One medical paediatric ward. SSPAU opened March 2012.	Yes	One hospital, in Melrose. In March 2012, two beds on the paediatric ward were nominated short stay beds.
NHS Dumfries and Galloway	22,478	2400 (9)	3 (2,4)	2.4 (0.9, 6.7)	One medical paediatric ward. SSPAU opened July 2011.	Yes	One hospital, in Dumfries. A 4-bedded short stay unit opened in 2011, as part of the medical ward and was open Mon-Fri 0900–2130 and only received paediatric emergency admission and had no overnight stays. A new hospital opened in 2017 (2 miles from the old hospital site) with a 12-bed SSPAU open Mon-Fri for 24 h and which accepts all medical, surgical, orthopaedic emergency admissions and elective theatre and ambulatory care.
NHS Fife	60,109	512 (117)	2 (1,4)	2.3 (0.8, 6.0)	One medical paediatric ward. SSPAU opened Jan 2011 when the paediatric department moved back to a new building on the original hospital site. To accommodate building, the paediatric department had been relocated for two years to another hospital less than one mile away.	Yes	One hospital, in Kirkcaldy. A general practitioner assessment bay was opened in Jan 2011 and called the SSPAU since November 2017. There are four beds in the unit.
NHS Forth Valley	49,594	1020 (49)	3 (2,4)	2.7 (0.9, 7.0)	One medical paediatric ward. SSPAU opened August 2011 when new hospital opened (on different site to old hospital).	Yes	One hospital, initially in Stirling but moved nine miles to Larbert in 2011. The SSPAU in the new hospital has 8 beds including two three-bedded bays and beds for examining patients.
NHS Grampian	91,660	3360 (27)	3 (2,4)	2.0 (0.7, 5.7)	Two hospitals (65 miles apart) each with a medical paediatric ward. SSPAU opened Jan 2004 when larger of two units moved to new hospital (on the same site as the old hospital).	Yes	Two hospitals, in Aberdeen and Elgin. A SSPAU opened in January 2004 at Royal Aberdeen Children’s Hospital. The unit has five cubicles and two five bedded wards. The unit was part of a new hospital which also opened January 2004. A second smaller unit at Dr. Gray’s Hospital in Elgin does not have an SSPAU
NHS Greater Glasgow and Clyde	179,365	453 (396)	2 (1,4)	1.8 (0.6, 5.1)	Two hospitals (10 miles apart) each with a medical paediatric unit. SSPAU opened in largest unit in 1996–7, and in Nov 2000 in the smaller unit. The larger unit has a paediatric emergency department (ED) and children seen and sent home in the ED are not considered an admission	No (SSPAU opened in larger unit before 2000)	Initially three hospitals, Yorkhill, Paisley and Inverclyde. Royal Glasgow Hospital for Sick Children (Yorkhill). A short stay ward opened in 1996–7, a six bed bay, a four bed bay and four cubicles. Royal Alexandra Hospital (Paisley). An acute assessment unit opened in November 2000. There were 5 beds in a bay and one cublicle. Inverclyde Royal Hospital admitted patients until November 2004, thereafter the inpatient service was centralised to RAH. There was an isolated short stay unit Monday to Friday with no overnight admissions until 2006.
NHS Highland	50,588	12,507 (4)	3 (2,4)	2.3 (0.8, 6.6)	One medical paediatric ward. SSPAU opened May 2016.	No (SSPAU opened after 2013)	One hospital, in Inverness. A five-bedded SSPAU opened in May 2016, next to the paediatric medical ward.
NHS Lanarkshire	111,306	883 (126)	2 (1,4)	2.3 (0.8, 6.7)	One medical paediatric ward. SSPAU opened Feb 2005.	Yes	
NHS Lothian	135,517	700 (194)	3 (2,4)	1.8 (0.6, 4.9)	Two hospitals (17 miles apart) each with a medical paediatric ward. SSPAU opened 2005 in smaller of two units only. The larger unit has a paediatric emergency department (ED) and children seen and sent home in the ED are not considered an admission.	No (SSPAU not opened in larger unit)	Two hospitals, one in Edinburgh and one in Livingstone. The hospital in Edinburgh (the larger of the two) has no SSPAU. The hospital in Livingstone opened a Paediatric Assessment Unit in 2005, a 6 bedded bay on the paediatric medical ward.
NHS Tayside	63,309	2986 (21)	3 (2,4)	2.8 (1.0, 7.4)	Two hospitals (22 miles apart) each with a medical paediatric ward. SSPAU opened 1997 in larger units and May 2004 in smaller unit when it moved to closing overnight.	No (SSPAU opened in larger unit before 2000)	Two hospitals, in Dundee and Perth. Ninewells hospital (Dundee). A paediatric assessment area was set up in late 1997, initially a bay in the ward then with a move to a new building in 2002 the unit was a separate area adjacent to the paediatric inpatient unit Perth Royal Infirmary (Perth). Inpatient services moved to Dundee in 2005 and an ambulatory care unit was maintained in Perth (open Mon-Fri 10 am-6 pm) where children with acute illnesses can be assessed.
Overall			3 (2,4)	2.3 (0.75, 6.25)		Data from 7 of 11 units included	

there were an additional 1305 admissions to three health boards without in patient paediatric services including 251 zero day admissions. ^a^data were available for ages 0–4, 5–9 and 10–14 year age bands

There were six health board regions with a single inpatient unit (i.e. hospital) and where an SSPAU opened between 2000 and 2013 and data from all these regions were included in the analysis, Table 1. Data were also included from a seventh health board (NHS Grampian) which had two inpatient units and where an SSPAU opened in the larger unit but not the smaller unit, Table 1. The was an eighth health board with an SSPAUs which opened before 2000 (NHS Greater Glasgow and Clyde). There were three health boards with no SSPAU open between 2000 and 2013 (NHS Tayside, NHS Lothian and NHS Highland), Table 1. Table 2 presents the sum of all admissions and zero day admissions to each health board, and also prevalence and change in number and change over time for zero day admissions.

**Table 2:** Details of childhood admissions to eleven health board areas in Scotland between January 2000 and December 2013, including number of all admissions and zero day admissions. A zero day admissions occurs when a child is admitted to hospital and discharged on the same day

NHS health board	Number of admissions 2000–2013	Number of zero day admissions (% of all admissions)	Annual prevalence of zero day admissions/1000 children	Number (% of zero day) admissions		Change in % zero day admission per month (Spearman rho)	SSPAU opened 2000–13
				Jan 2000	Jan 2013		
NHS Ayrshire and Arran	57,716	26,010 (45%)	37/1000	38 (16.1%)	428 (53.9%)	0.86 (*p* < 0.001)	Yes
NHS Borders	15,097	5138 (34%)	24/1000	17 (26.2%)	68 (66.7%)	0.61 (*p* < 0.001)	Yes
NHS Dumfries and Galloway	16,388	4532 (28%)	17/1000	6 (7.4%)	39 (35.1%)	0.77 (*p* < 0.001)	Yes
NHS Fife	34,462	13,683 (40%)	19/1000	71 (42.0%)	107 (13.3%)	−0.78 (*p* < 0.001)	Yes
NHS Forth Valley	42,361	17,855 (42%)	30/1000	43 (29.1%)	139 (46.1%)	0.39 (*p* < 0.001)	Yes
NHS Grampian	59,524	17,161 (29%)	17/1000	28 (11.9%)	108 (43.2%)	0.96 (*p* < 0.001)	Yes
NHS Greater Glasgow and Clyde	90,863	32,648 (36%)	15/1000	109 (20.7%)	71(34.7%)	0.73 (*p* < 0.001)	No (opened pre 2000)
NHS Highland	26,058	7530 (29%)	12/1000	22 (18.0%)	71 (39.9%)	0.71 (*p* < 0.001)	No
NHS Lanarkshire	77,373	38,608 (50%)	28/10000	31 (14.5%)	287 (57.3%)	0.56 (*p* < 0.001)	Yes
NHS Lothian	75,774	22,436 (30%)	14/10000	48 (14.5%)	168 (36.8%)	0.60 (*p* < 0.001)	No
NHS Tayside	73,482	34,665 (47%)	46/10000	116 (38.7%)	226 (54.5%)	0.73 (*p* < 0.001)	No
Overall	569, 098	220,517 (39%)	22/10000	529 (21.8%)	1479 (45.9%)	0.89 (*p* < 0.001)	

**Fig. 2:**
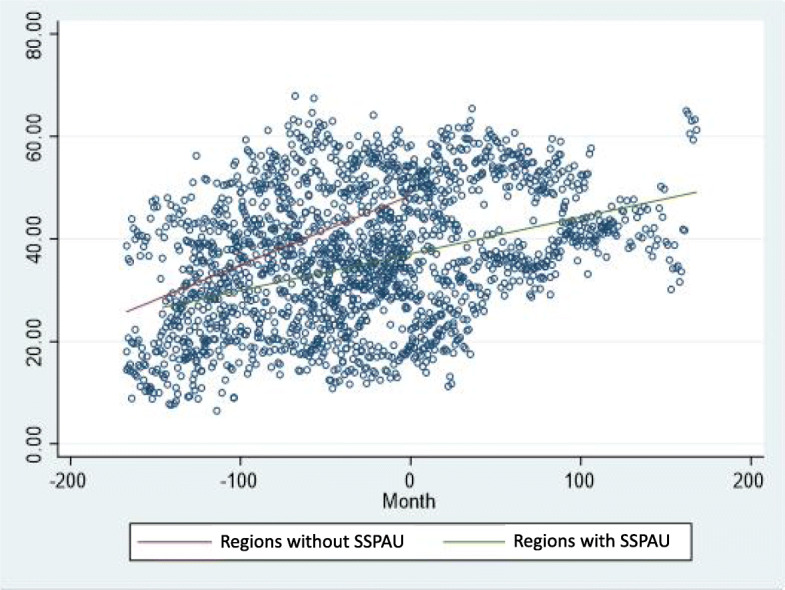
A scatter plot comparing the change over time in the percentage of all emergency admissions which were zero day admissions per month. The red line indicates the trend for hospitals with no short stay paediatric assessment unit (SSPAU) and the green line indicates hospitals which had an SSPAU. The interaction term between time (i.e. month) and SSPAU or no SSPAU was significant (*p* < 0.001)

The comparison of proportion of zero day admissions between the eight boards with an SSPAU (i.e. including NHS Greater Glasgow and Clyde) and the three boards without an SSPAU (i.e. NHS Highland, NHS Lothian and NHS Tayside) showed higher % zero day admissions in boards without SSPAU (mean difference 11%/month [95% CI 9, 13]). The rise in % zero day admissions was higher in boards where there was no SSPAU compared to those with an SSPAU (mean rise 0.6%/month [95% 0.04, 0.09]), Fig. 2.

**Fig. 3:**
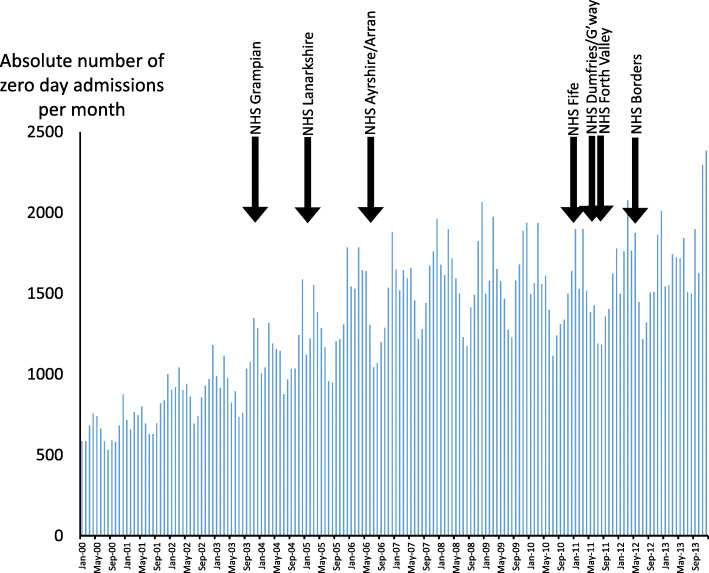
A bar chart indicating the monthly number of zero day admissions to all hospitals in Scotland between 2000 and 2013. The black arrows show when Short Stay Paediatric Assessment Units opened

Data from seven health boards where an SSPAU opened between 2000 and 2013 were considered in the time series analysis including: 302,921 acute medical admissions (mean 258/month, SD 148); 122,987 (105/month, SD 86) zero day admissions (41%), of whom 52,728 (35%) occurred before an SSPAU opened and 70,259 after an SSPAU opened (65%). The proportion of zero day admissions before and after opening of SSPAU is given in Table 3. Figure 3 shows the number of zero day admissions per month for all hospitals and indicates when the seven SSPAUs opened.

**Table 3:** The number of acute medical zero day admissions (“zero”, defined as being admitted and discharged on the same day and not readmitted within the same calendar month) and all acute medical admissions (“all”) and the relative change in the proportion of zero day admission before and after SSPAU opened. Data are provided for all seven health boards collectively and individually

	Total admissions 2000–2013		Admissions before SSPAU opened		Admissions after SSPAU opened		Percentage of zero day admissions		Relative increase in the proportion of zero day admissions after SSPAU opened
	all	zero	all	zero	all	zero	Before SSPAU opened	After SSPAU opened	
All seven health boards	302,921	122,987	148,017	52,728	154,904	70,259	35.6	45.4	27.5
NHS Ayrshire and Arran	57,716	26,010	20,544	5644	37,172	20,366	27.5	54.8	99.3
NHS Borders	15,097	5138	12,280	3708	2817	1430	30.2	50.8	68.2
NHS Dumfries and Galloway	16,388	4532	12,718	3194	3670	1338	25.1	36.5	45.4
NHS Fife	34,462	13,683	29,782	12,788	4680	895	42.9	19.1	−55.5
NHS Forth Valley	42,361	17,855	35,710	15,110	6651	2745	42.3	41.3	−2.4
NHS Grampian	59,524	17,161	13,686	2084	45,838	15,077	15.2	32.9	116
NHS Lanarkshire	77,373	38,608	23,297	10,200	54,076	28,408	43.8	52.5	19.9

### Relationship between SSPAU opening and proportion of zero day admissions

#### All health boards combined

**Fig. 4:**
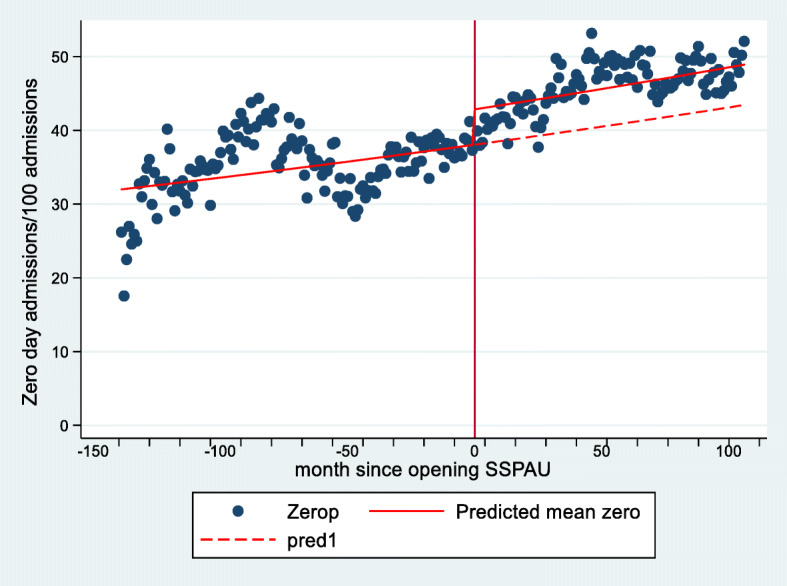
The proportion of zero day admission as a percentage of all acute admissions before and after opening of short stay paediatric assessment units in seven health boards (solid vertical line). The sloping solid red line indicates the trend in the proportion of zero day admissions before and after the SSPAU opened. The sloping dashed red indicates the trend in zero day admissions if the SSPAUs had not opened

The proportion of zero day admission rose by 28% between 2000 and 2013, Table 3. There was a 13% increase [95% CI 10, 15] in the proportion of zero day admissions after SSPAU opening, Table 4 and Fig. 4. The rise in admissions approximately 100 months before SSPAU opening (Fig. 4) was explained by admissions on one centre (NHS Fife). Independently of SSPAU opening there was a monthly increase of 0.12% [95% CI 0.11, 0.14] in the proportion of zero day admissions. The interaction term between time and SSPAU opening was significant, indicating that there was a change in the proportion of zero day admissions after SSPAUs opened.

**Table 4:** Results from the interrupted item series analysis. *p* < 0.001 unless stated. When the interaction term was insignificant, results from the main effects model are presented (i.e. NHS Borders, NHS Fife, NHS Forth Valley and NHS Grampian)

	Step change in percentage of zero day admissions relative to all admissions after SSPAU opened	Percentage change in zero day admissions per month between Jan 2000 and Dec 2013	Change in zero day admission after SSPAU open (Interaction term) (*p*-value)
All centres combined	+ 13 [10, 15]	+ 0.12 [0.11, 0.14]	*p* < 0.001
NHS Ayrshire and Arran	+ 71 [57, 86]	+ 0.18 [0.11, 0.26]	*p* < 0.001
NHS Borders	+ 14 [2, 27]	+ 0.53 [0.42, 0.64]	*p* = 0.398
NHS Dumfries and Galloway	*−*2 [−12, + 8]	+ 0.54 [0.44, 0.64]	*p* = 0.029
NHS Fife	−42 [−49, −36]	−0.29 [−0.37, −0.20]	*p* = 0.400
NHS Forth Valley	− 23 [− 27, 19]	+ 0.31 [0.27, 0.35]	*p* = 0.123
NHS Grampian	+ 24 [15, 32]	+ 0.63 [0.57, 0.69]	*p* = 0.325
NHS Lanarkshire	+ 6 [−1, + 15]	+ 0.15 [0.08, 0.22]	*p* < 0.001

#### Individual health boards

**Fig. 5:**
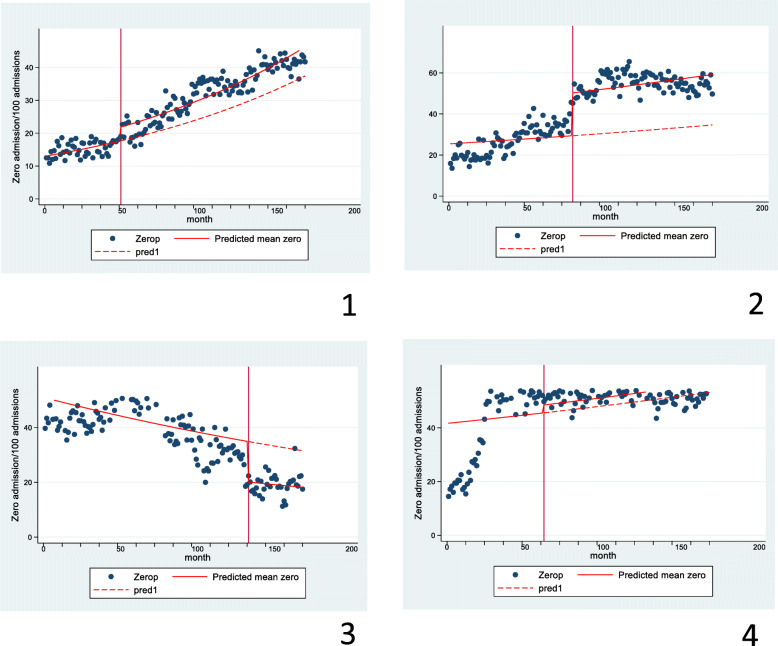
Trends in four health boards where zero day admissions rose (1, NHS Grampian and 2 NHS Ayrshire and Arran), fell (3, NHS Fife) or remained static (4, Lanarkshire) after an SSPAU opened. The number of months begins at 1 in Jan 2000 and finishes at 168 for December 2013. The vertical red line indicates when the SSPAU opened. The sloping solid red line indicates the trend in the proportion of zero day admissions before and after the SSPAU opened. The sloping dashed red indicates the trend in zero day admissions if the SSPAUs had not opened

For individual health boards (without considering the underlying trend in zero day admissions), the change in the proportion of zero day admissions after an SSPAU opened varied between a fall of 56% and a rise of 99%, Table 3. Figure 5 illustrates trends in four health boards where zero day admissions rose, fell or remained static after an SSPAU opened. Supplemental Figures S1, S2 and S3 present trends before and after SSPAU opened in the remaining three health boards. When considering the underlying trend in zero day admissions, the proportion of zero day admissions was higher in the period after an SSPAU opened than pre-opening in three health boards (NHS Ayrshire and Arran, NHS Borders and NHS Grampian), was lower in three health boards (NHS Fife, NHS Forth Valley and NHS Lanarkshire) and did not differ in a seventh (NHS Dumfries and Galloway), Table 4. In six health boards, the proportion of zero day admissions rose on a monthly basis independent of SSPAU opening and fell in a seventh health board (NHS Fife), Table 4. The interaction term between SSPAU opening and time was significant in four  health board (NHS Dumfries and Galloway, NHS Ayrshire and Arran and NHS Lanarkshire) and not significant for the remaining four health boards, Table 4.

### Sensitivity analyses

#### Removing initial month’s data for non-linear trends

Data were excluded from the analysis for the following months in these three boards: months 0–50 for Fife; 0–30 for Forth valley; and months 0–25 for Lanarkshire. Compared to the analysis without removing data, the association between opening an SSPAU and changes in admissions was comparable for NHS Fife and NHS Forth Valley but the interaction term became significant for both. For NHS Lanarkshire the results after removing the initial 25 month’s data found a neutral step change in admissions compared to the complete dataset but the rise in admissions and significant interaction term seen in the whole dataset were not apparent in the sensitivity analysis (Table S2 and Figure S4).

#### Analysing only 24 months before and after SSPAU opening

Here the smaller number of month’s studied yield different results compared to the main analysis. The magnitude of step change was smaller for all centres (individually and combined), remained significant for NHS Ayrshire and Arran (positive change) and NHS Forth valley (negative change), became significant for NHS Dumfries and Galloway and became non-significant for all centres, Table S3 and Figures S5 and S6. The interaction term became positive NHS Forth Valley but (compared to the main analysis) the previously significant interaction terms for all centres, NHS Ayrshire and Arran, NHS Dumfries and Galloway and NHS Lanarkshire were non-significant in the sensitivity analysis.

## Discussion

This study was designed to describe changes in the proportion of zero day admissions after an SSPAU was opened, and was undertaken in light of uncertainty in the literature. The impact on opening an SSPAU was heterogeneous between centres. The first analysis found that regions where an SSPAU was present had a lower absolute proportion of zero day admissions and a slower rise in proportion of zero day admissions compared to regions where no SSPAU was present. The second analysis, where only data from regions where an SSPAU opened between 2000 and 2013 found that the proportion of zero day admissions relative to all unscheduled medical paediatric admissions rose by 13% after SSPAUs opened, and by 15% over time due to other factors which we did not capture. Further inspection of the data showed that zero day admissions did not rise in all regions after an SSPAU opened, indeed admissions fell in some units. Collectively these findings suggest that in some circumstances opening an SSPAU may increase the proportion of zero day admissions, but in other circumstances may reduce zero day admissions. Our results are likely to be generalisable across the UK and to other similar healthcare systems.

Our sensitivity analyses explored the potential for non-linear changes in trends for admissions occurring many months before SSPAU opening to influence the apparent impact of the opening on admission trends, and also focussed on a shorter period of time before and after SSPAU opening. Predictably, given the reduced amount of data analysed and removing “outlying” data points, some results from sensitivity analyses differed from the main analysis (Table 4) but the overall message was retained, i.e. in different hospitals, opening an SSPAU either increased or reduced or made no difference to the proportion of zero day admissions.

There were only seven SSPAU opened in Scotland between 2000 and 2013 and this prevents a meaningful analysis of factors associated with rising or falling zero day admissions after an SSPAU opens. Factors such as opening an SSPAU as part of a new hospital build may be important to any impact on zero day admissions (the two boards with falling zero day admissions after SSPAU opened, i.e. NHS Fife and NHS Forth Valley, had moved to a new build); referral thresholds may differ between the period before the opening of a new in patient facility with integral SSPAU compared to the “old” facility. Also worthy of further evaluation is a possible inverse relationship between proportion of zero day admissions before an SSPAU opening and change in proportion of zero day admissions after the SSPAU opens (Table 2). The heterogeneous changes in zero day admissions after SSPAU open across Scotland may also be explained by regional differences in clinical referral pathways, and these drivers could be further explored in England where there is a greater number of hospitals and SSPAUs.

Our previous work [7] has described the rise in zero day admissions and here we add to these findings by exploring why this rise has occurred. Our findings indicate that although SSPAUs may have been opened in response to increasing zero day admissions, SSPAUs may in some regions have been an factor associated with the increase in zero day admissions. The National Audit Office state that “at least one-fifth of admissions could be managed effectively in the community” [13], and recent publications have suggested that as many as 50% of emergency presentations could have been managed in the community [14] and care pathways for infants could reduce many admissions [15]. The existence of a steady rise in zero day admissions which then rises after an SSPAU opens and accelerates thereafter suggests that additional community-based interventions may offer a safe and convenient method to manage at many of acute childhood illnesses currently referred to hospital. We have previously described fluctuation in the number of zero day admissions over the week, rising from Monday to a peak Friday followed by a falls to a nadir on Sunday [10], and this suggests that there is a “discretionary” element to zero day admissions which may reflect changing parental health seeking behaviour and availability of healthcare professionals.

There are many factors which are involved in the decision-making leading to an admission and these include: parental factors (education, experience, support); healthcare factors (availability of unscheduled services; paediatric expertise in unscheduled healthcare services; tertiary versus secondary hospital; referral pathways); SSPAU factors (size, staffing); child factors (severity of illness, past history, comorbidities, distance from hospital). Since these many factors were not considered in our analysis, our study cannot explain why zero day admissions changed in many units after an SSPAU opened.

There is evidence from a number of randomised controlled trials that care in the home for children is safe and no more expensive than providing care in the hospital setting [16], these trials are not all UK-based and are up to 20 years old so are not necessarily generalisable to the UK in 2018. An observational study in the UK has demonstrated that a model where a community nurse team managed children at home (mostly having been previously assessed on an SSPAU) was appreciated by parents and cost effective [17].

The fall in proportion of zero day admissions in two regions (Forth Valley and Fife) needs to be considered with some caution since in both of these regions the SSPAU opened 2011, which is late in the period 2000–2013 and these findings need to be replicated with extended follow up and more data. Alternatively, the management teams in Forth Valley and Fife may have learned lessons from SSPAUs which had opened earlier in other regions and created pathways which reduced zero day admissions. The SSPAU in one of two regions where there was no increase in the proportion of zero day admissions (Dumfries and Galloway) also opened in 2011 units and had the smallest number of zero day admissions, and this finding also needs to be replicated with longer follow up and more data. In contrast, the second unit with no increase in the proportion of zero day admissions after their SSPAU opened (Lanarkshire) opened in 2005 and had the second highest number of admissions of all regions, we are therefore confident that opening an SSPAU does not increase the proportion of zero day admissions in every setting.

Our study is limited in not being able to determine where short stay admissions were referred from i.e. general practitioners, out-of-hours staff to emergency department doctors. Admissions may previously have been seen in emergency departments, general practices or urgent care centres and from a total health system perspective it may be a good thing to have SSPAUs off-loading capacity from stretched emergency departments or general practices. However, having a short stay hospital admission is not necessarily in the child’s best interest and stretches paediatric resources. An additional limitation is that we do not have details of referral pathways in each health board; this information (if it exists) would be helpful in understanding why trends after SSPAUs opened. Community–based interventions lead by paediatric teams such as are proposed in the RCPCH Facing the Future [18] could reverse this shift in how children are cared for.

There are further limitations to our study. First, a zero day admission does not include all admissions which last less than 24 h, for example a child admitted at 10 am and discharged home at 3 am the following day would not be categorised as a zero day admission, but the large number of admissions included in our study will minimise the effect of this limitation. Second, since our outcome was proportion of zero day admissions relative to all admissions our analysis could not consider patient factors such as age and socioeconomic status. Third, in some centres (e.g. NHS Borders) data were available for less than 2 years after the SSPAU opened and more extended follow up in these centres might reveal different trends to those described here. A further limitation is that we have not considered how opening SSPAU might have affected zero day admissions stratified by diagnosis, and whilst there is evidence that an SSPAU might reduce admissions for certain conditions [2], our aim was to look at all admissions. One more limitation is that we did not include all data from all eleven units in a single analysis to consider both whether admissions changed after an SSPAU opened and whether trends in admissions differed over time between inpatient facilities who did and did not have an SSPAU. Finally where there were two inpatient facilities in a single health board region, our analysis pooled data from the two inpatient facilities even though only one had an SSPAU and this may have influenced the association between opening an SSPAU and zero day admissions.

## Conclusions

In summary we find that the impact of opening an SSPAU on the proportion of zero day admissions to hospital is not consistent. Although when all centres were considered there was an increase in zero day admissions after SSPAUs were opened, there was marked inter-centre differences with zero day admissions falling after an SSPAU opened in some centres. The population of children living in Scotland was lower in 2013 than in 2000 (868,921 versus 919,439 [7]) so any change in total admissions or zero day admissions cannot be due to a rise in population. Opening an SSPAU is a complex intervention which involves more than simply opening more beds. We found that an SSPAU often occurs as part of a new hospital opening and there was considerable heterogeneity in the design of the SSPAUs. Further understanding of the impact on zero day admissions after an SSPAU opens may provide other regions (and countries) with useful insights in managing children in the interface between community and hospital based services.

## Supplementary Information

**Additional file 1: Figure S1.** The trend (based on model) in zero day admission before opening of SSPAU in NHS Forth Valley (solid line) and counterfactual scenario as a dashed line in the post SSPAU opening period (interaction model). **Figure S2.** The trend (based on model) in zero day admission before opening of SSPAU in NHS Borders (solid line) and counterfactual scenario as a dashed line in the post SSPAU opening period (interaction model). **Figure S3.** The trend (based on model) in zero day admission before opening of SSPAU in NHS Dumfries and Galloway (solid line) and counterfactual scenario as a dashed line in the post SSPAU opening period (interaction model). **Table S1.** Descriptives available for analysis. **Table S2.** Results from the interrupted item series analysis where initial months (i.e. before SSPUA opened) were excluded from the analysis in three boards: months 0–50 for Fife; 0–30 for Forth valley; and months 0–25 for Lanarkshire. Step change and percentage change data are provided from the interaction model for NHS Forth Valley and NHS Fife (where the interaction term was significant) and from the main effects only model for NHS Lanarkshire. The values from the model including all data from table four are in italics and grey for comparison. **Figure S4.** The trend (based on model) in zero day in all NHS Fife (omitting data from months 0–50), Forth Valley (omitting data from months 0–30) and NHS Lanarkshire (omitting data form months 0–25). The solid line represents the trend and the dashed line is the counterfactual scenario (i.e. what would be expected to happen had the SSPAU not opened) from the interaction model. **Table S3.** Results from the interrupted item series analysis limited to 24 months before and after the SSPAU opened. *p* < 0.001 unless stated. Step change and percentage change data are provided from the main effects only model, except for NHS Forth Valley where the interaction term was significant. The values from the model including all data from table four are in italics and grey for comparison. **Figure S5.** The trend (based on model) in zero day admission in the 24 months before opening of SSPAU in all seven boards (solid line) and counterfactual scenario (i.e. what would be expected to happen had the SSPAU not opened) as a dashed line in the 24 months post the SSPAU opening (interaction model). **Figure S6.** The trend (based on model) in zero day admission in the 24 months before opening of SSPAU in NHS Forth Valley (solid line) and counterfactual scenario (i.e. what would be expected to happen had the SSPAU not opened) as a dashed line in the 24 months post the SSPAU opening (interaction model).

## Data Availability

The dataset used and analysed during the current study is available from the corresponding author on reasonable request.

## Abbreviations

GP: General Practitioner
NHS: National Health Service
RCPCH: Royal College of Paediatrics and Child Health
SSPAU: Short stay paediatric assessment unit
US: United States
