# Supplementary material for: The association between opening a short stay paediatric assessment unit and trends in short stay hospital admissions
Source: BMC Health Serv Res. 2021 May 29;21:523. doi: 10.1186/s12913-021-06541-x (PMC8164232; doi:10.1186/s12913-021-06541-x)
Supplement: Supplementary file 1 — Additional file 1: Figure S1. The trend (based on model) in zero day admission before opening of SSPAU in NHS Forth Valley (solid line) and counterfactual scenario as a dashed line in the post SSPAU opening period (interaction model). Figure S2. The trend (based on model) in zero day admission before opening of SSPAU in NHS Borders (solid line) and counterfactual scenario as a dashed line in the post SSPAU opening period (interaction model). Figure S3. The trend (based on model) in zero day admission before opening of SSPAU in NHS Dumfries and Galloway (solid line) and counterfactual scenario as a dashed line in the post SSPAU opening period (interaction model). Table S1. Descriptives available for analysis. Table S2. Results from the interrupted item series analysis where initial months (i.e. before SSPUA opened) were excluded from the analysis in three boards: months 0–50 for Fife; 0–30 for Forth valley; and months 0–25 for Lanarkshire. Step change and percentage change data are provided from the interaction model for NHS Forth Valley and NHS Fife (where the interaction term was significant) and from the main effects only model for NHS Lanarkshire. The values from the model including all data from table four are in italics and grey for comparison. Figure S4. The trend (based on model) in zero day in all NHS Fife (omitting data from months 0–50), Forth Valley (omitting data from months 0–30) and NHS Lanarkshire (omitting data form months 0–25). The solid line represents the trend and the dashed line is the counterfactual scenario (i.e. what would be expected to happen had the SSPAU not opened) from the interaction model. Table S3. Results from the interrupted item series analysis limited to 24 months before and after the SSPAU opened. p < 0.001 unless stated. Step change and percentage change data are provided from the main effects only model, except for NHS Forth Valley where the interaction term was significant. The values from the model including all data from t [file 12913_2021_6541_MOESM1_ESM.docx]

Steve Turner^1^, Edwin-Amalraj Raja^2^

The association between opening a short stay paediatric assessment unit and trends in short stay hospital admissions

^1^Child Health, University of Aberdeen

^2^ Medical Statistics Team, University of Aberdeen

Figure S1. The trend (based on model) in zero day admission before opening of SSPAU in NHS Forth Valley (solid line) and counterfactual scenario as a dashed line in the post SSPAU opening period (interaction model).

Figure S2. The trend (based on model) in zero day admission before opening of SSPAU in NHS Borders (solid line) and counterfactual scenario as a dashed line in the post SSPAU opening period (interaction model).

Figure S3. The trend (based on model) in zero day admission before opening of SSPAU in NHS Dumfries and Galloway (solid line) and counterfactual scenario as a dashed line in the post SSPAU opening period (interaction model).

| **Variable name** | **Description** |
| --- | --- |
| PatientID | Anon patient ID |
| SEX | Sex (1=male, 2=female) |
| AGE_IN_YEARS | Age at admission (years) |
| AGE_IN_MONTHS_ADDITIONAL | Additional months to AGE_IN_YEARS (months) |
| SIGNIFICANT_FACILITY | Significant facility (see SMR01 coding tab) |
| ADMISSION_TYPE | Type of admission (see SMR01 coding tab) |
| ADMISSION_DAY_OF_WEEK | Admission day of week (MON, TUE, etc.) |
| ADMISSION_MONTH | Admission month of year (1=Jan, 2=Feb etc.) |
| ADMISSION_YEAR | Admission year |
| DISCHARGE_DAY_OF_WEEK | Discharge day of week (MON, TUE, etc.) |
| LENGTH_OF_STAY | Length of stay (days) |
| MAIN_CONDITION | Main condition (ICD10) |
| MAIN_CONDITION_DESCRIPTION | Main condition description |
| DISCHARGE_TYPE | Discharge type (see SMR01 coding tab) |
| INPATIENT_DAYCASE_IDENTIFIER | I=Inpatient, D=Daycase |
| scsimd2012quintile | SIMD 2012 Scotland level quintile (1=most deprived; 5=least deprived) |
| hbsimd2012quintile | SIMD 2012 Health Board level quintile (1=most deprived; 5=least deprived) |
| HBTREAT | Health Board of treatment (see SMR01 coding tab) |
| SPECIALTY | AF=Paediatrics, CA=Paediatric Surgery, D8=Paediatric Dentistry |

Table S1. Descriptives available for analysis.

| **Admission type:** | **ISD Scotland Data Dictionary - Admission type** |
| --- | --- |
| **Code** | **Description** |
| Emergency Admission |  |
| 30 | Emergency Admission, no additional detail added |
| 31 | Patient Injury - Self Inflicted (Injury or Poisoning) |
| 32 | Patient Injury - Road Traffic Accident (RTA) |
| 33 | Patient Injury - Home Incident (including Assault or Accidental Poisoning in the home) |
| 34 | Patient Injury - Incident at Work (including Assault or Accidental Poisoning at work) |
| 35 | Patient Injury - Other Injury (inc. Accidental Poisoning other than in the home) - not elsewhere classified |
| 36 | Patient Non-Injury (e.g. stroke, MI, Ruptured Appendix) |
| 38 | Other Emergency Admission (including emergency transfers) |
| 39 | Emergency Admission, type not known |
|  |  |
| Other Admission |  |
| 40 | Other admission types, no additional detail added |
| 42 | Maternity Admission (SMR02 only) |
| 48 | Other |
|  |  |
| Routine Admission |  |
| 10 | Routine Admission, no additional detail added |
| 11 | Routine elective (i.e. from waiting list as planned, excludes planned transfers) |
| 12 | Patient admitted on day of decision to admit, or following day, not for medical reasons, but because suitable resources are available |
| 18 | Planned transfers |
| 19 | Routine Admission, type not known |
|  |  |
| Urgent Admission |  |
| 20 | Urgent Admission, no additional detail added |
| 21 | Patient delay (for domestic, legal or other practical reasons) |
| 22 | Hospital delay (for administrative or clinical reasons e.g. arranging appropriate facilities, or test to be carried out, specialist equipment, etc.) |
|  |  |
|  |  |
| **Discharge type:** | ISD Scotland Data Dictionary - Discharge type |
| **Code** | **Description** |
| Regular Discharge |  |
| 10 | Regular discharge, no additional detail added |
| 11 | Discharge from NHS inpatient/daycase care |
| 12 | Transfer within the same Provider Unit |
| 13 | Transfer to other Provider Unit |
| 14 | Patient given Extended Pass/Leave of Absence |
| 15 | Patient discharged by Mental Welfare Commission (SMR04 only) |
| 16 | Patient discharged under Community Care Order (SMR04 only) |
| 18 | Other type of regular discharge |
| 19 | Regular discharge, type not known |
|  |  |
| Irregular Discharge |  |
| 20 | Irregular Discharge, no additional detail added |
| 21 | Patient discharged himself/herself against medical advice |
| 22 | Patient discharged by relative |
| 23 | Patient absconded from detention (Record type 04 only) |
| 28 | Other type of irregular discharge |
| 29 | Irregular discharge, type not known |
|  |  |
| Death |  |
| 40 | Death, no additional detail added |
| 41 | Death - Post Mortem |
| 42 | Death - No Post Mortem |
| 43 | Death – Whilst on Pass |
|  |  |
|  |  |
| **Significant facility:** | **ISD Scotland Data Dictionary - Significant facility** |
| **Code** | **Description** |
| 11 | Other (inc. the Clinical Facilities of Standard Specialty Ward 1K, Day Bed Unit 1J) |
| 13 | Intensive Care Unit |
| 14 | Cardiac Care Unit |
| 16 | Children's Unit |
| 17 | Accident & Emergency (A&E) Ward |
| 18 | Ward for Younger Physically Disabled |
| 19 | Spinal Unit |
| 1A | Geriatric Orthopaedic Rehabilitation Unit (GORU) |
| 1B | Rehabilitation Ward (except GORU) |
| 1C | Burns Unit |
| 1D | Geriatric Assessment Unit |
| 1E | Long Stay Unit for Care of the Elderly |
| 1F | Convalescent Unit |
| 1G | Palliative Care Unit |
| 1H | High Dependency Unit |
| 1L | Adolescent Unit |
| 1M | Transplant Unit |
| 1N | Mother and Baby Unit |
| 1P | Stroke Unit |
| 1Q | Secure Psychiatric Inpatient Facility |
| 1R | Intensive Psychiatric Care Unit (IPCU) |
| 1S | Long Stay Unit - Mental Health |
| 1T | Psychiatric Rehabilitation Unit (PRU) |
| 31 | Outpatient Department |
| 32 | Accident & Emergency Department |
| 33 | Day Hospital |
| 34 | Health Centre |
| 35 | GP Surgery Premises |
| 36 | Patient's home |
| 37 | Other Community Premises |
| 38 | Rapid Access Chest Pain Clinic |
| 39 | Ambulatory Care Hospitals |
| 40 | Acute Assessment Unit (AAU) |
|  |  |
|  |  |
| **Health Board of treatment** |  |
| **Code** | **Description** |
| S08000001 | NHS Ayrshire & Arran |
| S08000002 | NHS Borders |
| S08000003 | NHS Dumfries & Galloway |
| S08000004 | NHS Fife |
| S08000005 | NHS Forth Valley |
| S08000006 | NHS Grampian |
| S08000007 | NHS Greater Glasgow & Clyde |
| S08000008 | NHS Highland |
| S08000009 | NHS Lanarkshire |
| S08000010 | NHS Lothian |
| S08000011 | NHS Orkney |
| S08000012 | NHS Shetland |
| S08000013 | NHS Tayside |
| S08000014 | NHS Western Isles |

Table S2. Results from the interrupted item series analysis where initial months (i.e. before SSPUA opened) were excluded from the analysis in three boards: months 0-50 for Fife; 0-30 for Forth valley; and months 0-25 for Lanarkshire. Step change and percentage change data are provided from the interaction model for NHS Forth Valley and NHS Fife (where the interaction term was significant) and from the main effects only model for NHS Lanarkshire. The values from the model including all data from table four are in italics and grey for comparison.

|  | Step change in percentage of zero day admissions relative to all admissions after SSPAU opened | Percentage change in zero day admissions per month between Jan 2000 and Dec 2013 | Change in zero day admission after SSPAU open (Interaction term)  Direction (p-value) |
| --- | --- | --- | --- |
| NHS Fife | -29 [-40, -15] | -0.94 [-1.07, -0.81] | Positive (p=0.006) |
|  | *-42 [-49, -36]* | *-0.29 [-0.37, -0.20]* | *Positive (p=0.400)* |
| NHS Forth Valley | -23 [-32, -14] | +0.81 [+0.15, +1.47] | Positive (p=0.016) |
|  | *-23 [-27, -19]* | *+0.31 [-0.27, +0.35]* | *Positive (p=0.123)* |
| NHS Lanarkshire | +0.3 [-3, +4] | -0.01 [-0.04, +0.03] | Negative (p=0.535) |
|  | *+6 [-1, +15]* | *+0.15 [0.08, 0.22]* | *Negative (p<0.001)* |

Figure S4. The trend (based on model) in zero day in all NHS Fife (omitting data from months 0-50), Forth Valley (omitting data from months 0-30) and NHS Lanarkshire (omitting data form months 0-25). The solid line represents the trend and the dashed line is the counterfactual scenario (i.e. what would be expected to happen had the SSPAU not opened) from the interaction model.


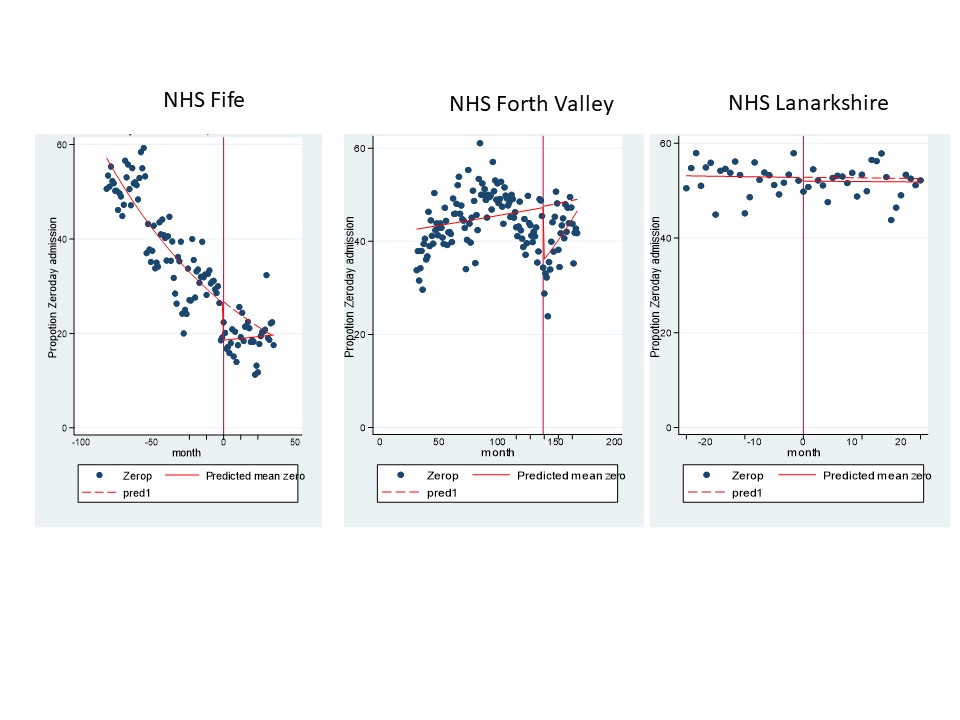


Table S3. Results from the interrupted item series analysis limited to 24 months before and after the SSPAU opened. p<0.001 unless stated. Step change and percentage change data are provided from the main effects only model, except for NHS Forth Valley where the interaction term was significant. The values from the model including all data from table four are in italics and grey for comparison.

|  | Step change in percentage of zero day admissions relative to all admissions after SSPAU opened | Percentage change in zero day admissions per month between Jan 2000 and Dec 2013 | Change in zero day admission after SSPAU open (Interaction term)  Direction (p-value) |
| --- | --- | --- | --- |
| All centres combined | +1 [-3, +6] | +0.38 [+0.22, +0.53] | Positive (p=0.152) |
|  | *+13 [10, 15]* | *+0.12 [ 0.11, 0.14]* | *Positive (p<0.001)* |
| NHS Ayrshire and Arran | +25 [+14, +37] | +0.96 [+0.66, +1.27] | Positive (p=0.627) |
|  | *+71 [+57, +86]* | *+0.18 [+0.11, +0.26]* | *Negative (p<0.001)* |
| NHS Borders | -4[-16, +10] | +0.57 [-0.15, 1.29] | Negative (p=0.143) |
|  | *+14 [2, 27]* | *+0.53 [+0.42, +0.64]* | *Negative(p=0.398)* |
| NHS Dumfries and Galloway | +23 (+1, +49] | +0.21[-0.81, 0.39] | Positive (p=0.951) |
|  | *-2 [-12, +8]* | *+0.54 [+0.44, +0.64]* | *Positive (p=0.029)* |
| NHS Fife | -24 [-38, -8] | -0.94 [-1.27, -0.66] | Positive (p=0.744) |
|  | *-42 [-49, -36]* | *-0.29 [-0.37, -0.20]* | *Positive (p=0.400)* |
| NHS Forth Valley | -19 [-29, -7] | +0.06 [-0.69, 0.56] | Positive (p=0.001) |
|  | *-23 [-27, -19]* | *+0.31 [0.27, 0.35]* | *Positive (p=0.123)* |
| NHS Grampian | +14 [-0.2, +29] | +0.71 [+0.25, +1.18] | Positive ( p=0.862) |
|  | *+24 [+15, +32]* | *+0.63 [+0.57, +0.69]* | *Positive (p=0.325)* |
| NHS Lanarkshire | -1 [-7, +4] | -0.02 [-0.23, 0.18] | Positive (p=0.959) |
|  | *+6 [-1, +15]* | *+0.15 [0.08, 0.22]* | *Negative (p<0.001)* |

Figure S5. The trend (based on model) in zero day admission in the 24 months before opening of SSPAU in all seven boards (solid line) and counterfactual scenario (i.e. what would be expected to happen had the SSPAU not opened) as a dashed line in the 24 months post the SSPAU opening (interaction model).

Figure S6. The trend (based on model) in zero day admission in the 24 months before opening of SSPAU in NHS Forth Valley (solid line) and counterfactual scenario (i.e. what would be expected to happen had the SSPAU not opened) as a dashed line in the 24 months post the SSPAU opening (interaction model).
